# Counting dots or counting reads? Complementary approaches to estimate virus-to-microbe ratios

**DOI:** 10.1038/s41396-023-01468-z

**Published:** 2023-08-18

**Authors:** Simon Roux, Jennifer R. Brum

**Affiliations:** 1 DOE Joint Genome Institute, Lawrence Berkeley National Laboratory, Berkeley, CA USA; 2 Department of Oceanography and Coastal Sciences, Louisiana State University, Baton Rouge, LA USA

**Keywords:** Environmental microbiology, Bacteriophages

“How many viruses are there in the environment, compared to cells ?”. This deceptively simple question remains challenging to address today, yet because it relates to fundamental processes and characteristics of viral communities, even the imperfect approximations available have been critical for the field of viral ecology. Most notably, early quantitative observations reporting a high abundance of virus-like particles in aquatic environments [1, 2] put a bright spotlight on viruses in the microbial ecology field, and spurred many to investigate the possible roles and impacts of these viruses [3, 4]. Admittedly, these also led to some redundancy in the introduction of many viral ecology publications, with statements such as “viruses are the most abundant entities” or “viruses outnumber cells by 10 to 1” almost systematically included (about 1550 hits in Google Scholar as of May 12, 2023, a trend to which we ourselves and almost every researcher in the field contributed, for better or worse).

This “virus to cell” ratio, also referred to as the virus-to-microbe ratio (VMR or VTM), virus-to-bacterium ratio (VBR), or virus-to-prokaryote ratio (VPR), is often reported as a key ecological metric, and typically derives from counts of viruses and cells using transmission electron microscopy (TEM), epifluorescence microscopy (EFM) or flow cytometry (FCM). Among these, EFM counts have been the most widely used as they combine a relatively high throughput and an ability to distinguish even small viruses from background noise [5]. These abundances of viruses, cells, and their ratio are some of the basic pieces of information used to estimate the potential impacts of viruses on ecosystem food webs and microbial processes, alongside other key metrics such as frequency of visibly infected cells or bacterial production. Yet, the relevance and usefulness of these all-embracing counts of all observable virus-like particles and all microbial cells in a sample remain questionable.

In aquatic environments, a large-scale meta-analysis indicated that, while a 10:1 ratio could represent a median value for some ecosystems, VMRs typically span across ~ 2 to 3 orders of magnitude so that “a 10:1 model has either limited or no explanatory power” [6]. A similar pattern of virus-like particles being overall more abundant than microbial cells, but with high sample-to-sample variation in the specific ratio, was reported for other environments [4]. Beyond the over-generalization of the 10 to 1 ratio in the literature, counts of virus-like particles are not without biases and limitations [7, 8]. Several types of structures can be counted as “virus-like particles” yet not be infectious viruses, including defective virions, Gene Transfer Agents, and in some cases DNA-containing vesicles or minerals. At the same time, some genuine viruses can be challenging to identify and count, including ssDNA and RNA viruses for which standard dyes are not always efficient, and large viruses that can be confounded with microbial cells. Virus-like particle counts can thus be both under- and over-estimated, with no clear way to better constrain this uncertainty using current methods. Consequently, alternative and complementary approaches enabling estimation of virus:microbe ratios, or even better virus:host ratios, are highly desirable and a topic of active research.

Meanwhile, these last 15 years have seen a rapid transformation of the viral ecology field with the fast rise of metagenomics. Large genome catalogs for uncultivated viruses obtained from metagenomes already provided invaluable information regarding their functional diversity, distribution across ecosystems, and dynamics through space and time [9]. Given the large amount of metagenome data already available and likely to be generated in the near-future, the prospect of leveraging metagenome-assembled genomes to estimate VMR is very appealing. Yet, the compositional nature of metagenomic data along with uncertainties around viral sequence detection methods have so far limited such attempts, and it is not entirely clear if and how much metagenomes can be used in this area.

In a new study, López-García et al. try to tackle this challenge by formally establishing a metagenome-based virus to (cellular) microbe ratio (mVMR), and compare it to microscopy-based counts across several ecosystems and sample types including bulk samples, cellular fractions, and viral fractions [10]. The mVMR metric relies on updated collections of single-copy marker genes enabling the detection of most of the major known viral, bacterial, and archaeal groups, with the ratio of abundance for viral and host markers used as a proxy for the virus:cell ratio in the original sample. When comparing mVMR estimations to epifluorescence-based counts (“fVMR”) for a set of aquatic samples and size fractions, both metrics typically ranged between 1:1 and 10:1 viruses to cells, yet, intriguingly, the overall correlation was limited between the two approaches. In freshwater environments, mVMR was more than twice that of fVMR, while the opposite was true in some marine and hypersaline samples. These discrepancies may be due to technical limitations, but could also reflect biological differences between these ecosystems, including e.g., the number and abundance of “non-viral virus-like particles” (likely counted with fVMR but not mVMR), as well as the abundance and frequency of viral genomes integrated in host genomes with little to no virion production (likely counted in mVMR but not fVMR).

Applied to a broader range of samples and ecosystems, mVMR typically range between 1:1 and ~10:1 with a relatively high sample-to-sample variation. Some broad ecosystem trends were nevertheless apparent, with higher mVMR in aquatic ecosystems compared to soil/sediments and animal-associated microbiomes. López-García et al. also illustrate another potential advantage of the mVMR approach by providing an estimated relative abundance of individual taxa within the aggregated “virus” and “microbe” counts for each sample, leveraging the fact that single-copy genes can also be used as taxonomic markers. This larger analysis illustrates both the promises and the remaining challenges of mVMR as a viral ecology metric. On one side, the prospect of leveraging the ever-growing number of public metagenomes, along with the possibility to estimate mVMR in a lineage-specific way to more closely reflect virus:host relationships, are tantalizing and would enable a much larger and in-depth investigation of virus:host dynamics across microbiomes. On the other hand, it is still complicated at this point to determine whether mVMR applied in this way is a “superior” metric, i.e., more accurate and less biased than microscopy counts.

Several technical limitations could lead to both under- and over-estimation of mVMR. Publicly available metagenomes most often target cellular size fractions, and may miss a significant number of viruses including (i) viral genomes encapsidated in virions, (ii) viruses not represented in current collections of single-copy marker genes, and (iii) ssDNA and RNA viruses not captured by standard library preparation protocols. While the most common dsDNA viruses are now most likely well captured by single-copy marker genes, a comprehensive mVMR would have to rely on integrated sampling across size fractions and a combination of DNA and RNA libraries, which would not be available for the majority of publicly available data. Meanwhile, mVMR counts will also include elements not typically considered in the VMR metric, e.g., dormant viruses that do not produce virions and/or kill their host, or virus-like machinery encoded by microbes and including homologs of virus marker genes. Hence, with the potential for relatively large under- and over-estimation, it is still difficult to get a sense for how accurate the mVMR metric is.

Ultimately, mVMR and fVMR appear to provide orthogonal perspectives on a highly complex biological process with different technical biases and limitations. On a bad day, a viral ecologist would only see two flawed metrics with unknown intervals of confidence that cannot be trusted. But on a good day, these two approaches can easily be seen as highly complementary, and the addition of mVMR next to fVMR could provide a much-needed additional window into virus:host interactions in microbiomes. With more controlled studies of known consortia to better evaluate methodological biases, advances in “quantitative sequencing” with the use of e.g., artificial spike-ins or high-throughput single-cell approaches, and more studies providing paired microscopy- and sequencing-based observations, we believe that the limitations around fVMR and mVMR will progressively be reduced, finally enabling viral ecologists to be more confident in these measurements. For better or worse, however, since both fVMR and mVMR seem to suggest that viruses typically outnumber cells across diverse samples and microbiomes, we anticipate to keep reading variants of the “viruses are the most abundant …” introduction statement for the foreseeable future.

## References

[1] Bergh O, Børsheim KY, Bratbak G, Heldal M (1989). High abundance of viruses found in aquatic environments. Nature.

[2] Proctor LM, Fuhrman JA (1990). Viral mortality of marine bacteria and cyanobacteria. Nature.

[3] Suttle CA (2007). Marine viruses—major players in the global ecosystem. Nat Rev Microbiol.

[4] Williamson KE, Fuhrmann JJ, Wommack KE, Radosevich M (2017). Viruses in soil ecosystems: an unknown quantity within an unexplored territory. Annu Rev Virol.

[5] Noble RT, Fuhrman JA (1998). Use of SYBR Green I for rapid epifluorescence counts of marine viruses and bacteria. Aquat Microb Ecol.

[6] Wigington CH, Sonderegger D, Brussaard CPD, Buchan A, Finke JF, Fuhrman JA (2016). Re-examination of the relationship between marine virus and microbial cell abundances. Nat Microbiol.

[7] Brum JR, Sullivan MB (2015). Rising to the challenge: accelerated pace of discovery transforms marine virology. Nat Rev Microbiol.

[8] Turzynski V, Monsees I, Moraru C, Probst AJ (2021). Imaging techniques for detecting prokaryotic viruses in environmental samples. Viruses.

[9] Sommers P, Chatterjee A, Varsani A, Trubl G (2021). Integrating viral metagenomics into an ecological framework. Annu Rev Virol.

[10] López-García P, Gutiérrez-Preciado A, Krupovic M, Ciobanu M, Deschamps P, Jardillier L, et al. Metagenome-derived virus-microbe ratios across ecosystems. ISME J. 2023:1–12. 10.1038/s41396-023-01431-y. 10.1038/s41396-023-01431-y PMC10504350 37169871

